# An Essay on Erysipelas, Read before the Cincinnati Medical Society, 1832

**Published:** 1832

**Authors:** Edwin A. AtLee


Art. IV.—*An Essay on* Erysipelas, *read before the Cincinnati Medical Society*, 1832. By Edwin A. At Lee, M. D.

We were entertained and edified on the last evening of our meeting, with an Essay on the characteristic differences between animate and inanimate nature. In that essay, it seemed very satisfactorily proved, that even in creatures organized and vivified, the laws of chemical affinity were not altogether suspended; while at the same time the learned author admitted that the principle of vitality held over those laws an undisputed sway.

Some observations also made on vitality itself, were not without interest, notwithstanding their seeming obscurity, caused principally by the reluctance which almost all scientific men entertain, against having recourse, except indirectly, to a first cause, the inspirer and modifier of life, the essence and potency of which He alone is, and must be: for it is reasonable to believe, that all creation exists and is sustained by what has very properly been denominated an *Influx* from the Creator, which, although one and the same, presents appearances diversified according to the differently constituted forms which are in existence. This influent Principle is demonstrated to any one who attends to the various instruments of sound, called wind instruments, which all receive the *ærial influx* at *one* orifice, but emit sounds indefinitely varying according to their specific forms, and the varied manipulations of the musician.

But I forbear, lest I be deemed *visionary;* and must descend from my metaphysical eminence, and enter on the less speculative, but not less appropriate subject, selected for this evening, which is a plain, practical essay on ERYSIPELAS.

The disease to which this name has been given, is one of peculiar character, which nosologists have agreed to place among the Exanthemata, or febrile diseases which effloresce upon the surface of the body. It may very properly be distinguished from all others by its *aptitude to extend over the skin*, or to bring into consent the parts in the neighborhood of that in which it originates. Hence the name *Erysipelas*, derived from the Greek, *eruo* to *draw* or attract, and *pelas* adjoining. It is known also by the names, *Rose*, *St. Anthony's Fire*, *Ignis Sacer.*

In Cullen's nosology it is a genus of disease in the class, *Pyrexiæ*, characterized by frequency of pulse after cold shivering, with increase of heat, and especially, among other impaired functions, with diminution of strength.

Of this disease Cullen gives two species, *Erysipelas Vesiculosum*, with *large blisters;* second, *Erysipelas Phlyctenodes*, with small blisters, called also *shingles*, from cingula, a girth, because it girds or surrounds the body. These definitions are for the most part correct, but that Erysipelas does sometimes manifest itself without the blisters, I entertain no doubt.

Dr. Good mentions two varieties of this disease, namely the *local* and *erratic;* but, with deference to so great a name, I am disposed to think his division incorrect, since the very title of the affection precludes the idea of fixed locality, by plainly indicating its wandering character.

Erysipelas is an inflammatory disorder, either of the skin externally or the mucous membrane internally. On the skin it may make its appearance in several places simultaneously, and is known by a deep red color, painful and burning heat, and swelling. The parts of the body most liable to attack are the extremities and face, and when the latter is the seat of disease, it is often preceded, for two or three days, by a tenderness and soreness of the bones underneath the part about to effloresce, as if they had suffered severe contusion.

It is supposed by some writers that women are more subject to Erysipelas than men are; and those of irritable habit, and robust and plethoric constitution more liable than others. My experience enables me to agree with authors of this sentiment. I have supposed also, that persons of gouty diathesis are more exposed to its attacks than those of other peculiarities of constitution; especially do such seem to be obnoxious, in whom, although inheriting the diathesis, arthritis, strictly speaking, has never manifested itself; such also, whose system has been reduced by frequent paroxysms of gout, so as to be no longer susceptible of them.

When speaking of the parts most subject to Erysipelas, the extremities and face were specified; but every part of the body is liable to it, and very young children, as well as adults, are often its victims.

The *causes* are such as are capable of exciting inflammation, namely external injuries, the application of stimulants, obstructed perspiration, exposure to cold, and certain derangements of function in the alimentary canal, some of which are induced by intemperance in eating and drinking. It is known also to follow suppressed evacuations of every kind; and is thought by some writers of celebrity to be sometimes epidemical: I am not disposed to doubt that particular states of the atmosphere may render it so.

It were happy for our profession if the *diagnosis* of Erysipelas were as free from obscurity as its etiology. There seems to have been too great a fondness, sometimes unwarranted by experience, for fabricating distinctions without differences, in the characteristic signs of the disease in question. Even practitioners of merited eminence, are viewed by my comparatively superficial intellect, as having greatly exceeded the limits of correct pathological classification. Thus Mr. Lawrence divides the varieties of Erysipelas into three, the *simple*, the *œdematous*, and the *phlegmonous*. Why the last should have been included is difficult to conjecture, since that species of inflammation is marked by a throbbing tumor, pointing to some definite outlet for its *purulent* contents; whereas Erysipelas, so long as it is confined to its proper sphere of action, the skin, never shews any disposition to suppurate; never manifests, like phlegmon, the hard, circumscribed tumor caused by effusion of coagulable lymph immediately beneath the skin. And even when Erysipelas penetrates the cellular texture, and thus throws out a kind of pus, it never becomes circumscribed, but runs into sinuosities between the muscles, like a quagmire, an instance of which occurred in my practice, wherein the left arm was thus affected, nearly from the shoulder to the elbow, and the matter was evacuated by a seaton and bandage. And in the disease under consideration, the termination by resolution distinguishes it from phlegmon, for this never desquamates.

It would, in my judgment be best always to designate the subject of this essay by the two peculiarities, of active and extensive *spreading*, and *scaly resolution*, than by accidental or adventitious marks induced by certain states of the patient or of the atmosphere. We are assisted in our diagnosis by the following facts, namely; phlegmonous inflammation uniformly commences in a part comparatively deep-seated, and points and escapes at the surface; while Erysipelas commences at the surface and rarely penetrates beyond it. Phlegmonous Inflammation never vesicates, but Erysipelas mostly does. The color of Phlegmon is bright or blushing red; that of Erysipelas dark red or yellowish, and its termination, when severe, is in gangrene; but phlegmon is, I believe, not known to terminate thus, except when it attacks the viscera. Independently of these distinguishing characteristics, there are others referred to by authors, and which may or may not be confirmed by experience; such as the *contagiousness* of Erysipelas, if Dr. *Wells's* facts *be* facts, and its capability of being communicated by *innoculation*, if Dr. Willan be correct.

In relation therefore to this disease, it is probably *sui generis*, and there does not appear to be any necessity for confounding it with any other. I proceed to an enumeration of its Symptoms. When not of the mildest form, it is commonly preceded by chilliness, lassitude, and pain, alternating with heat. These symptoms continue for a day or two, when a burning and itching is felt in the place invaded. If this be the face the inflammation shows itself on one side, commencing behind and below the ear, which it brings into consent, and spreading speedily over the cheek, nose, eyelids, forehead, scalp, and even down the back of the neck to between the scapulæ. Sometimes it commences at the root of the nose, and expatiates thence until the neighboring parts are involved as above described. Any raspatory irritation at this time hastens its career.

Whatever be the intensity of the redness, which is various in degree, the pressure of the finger causes the redness to disappear, and so soon as the finger is removed, that returns. If the progress of the disease be not now arrested, there soon appear vesicles filled with a fluid at first limpid then yellowish, which break, and the fluid escaping dries and falls off in scales. This last is the termination of the mild form of the disease, attended with little fever or other general affection. But in severe cases when inflammatory symptoms run high, there is great pain in the head and back, much thirst, heat and restlessness, with swelling of the affected part. The pulse becomes small and frequent, sometimes hard and tense, and about the fourth day the vesicles arise, the fluid of which, instead of being emptied out as usual, becomes viscid, and adheres firmly to the skin. Sometimes, but not frequently, the blisters degenerate into obstinate and even dangerous ulcers; and in some cases the eruption, which in general soon disappears, will continue for fourteen days or longer. In these the inflammation and swelling are excessive, and accompanied with considerable fever and delirium.

It is remarked by Dr. Dewees, that when but one side of the face is affected, "a line of demarcation is drawn from the forehead to the chin." Although I do not recollect having seen this fact mentioned by any other writer, I am inclined to give it my assent, having good reason for relying on the talent for observation, the experience, and *integrity* of that estimable practitioner, whose observations are generally of a character not lightly to be disputed or impeached. So far as the characteristic sign just mentioned relates to the *face*, my experience as a *patient* induces me to admit its correctness; but although I have twice suffered under severe attacks of Erysipelas of the face and head, I am not prepared to say that this line of separation does always extend along the *scalp*, for mine was wholly affected, yet in greatest degree on the side corresponding with the diseased face.

For the most part, after having run its course, it does not return till after some months or a year; but sometimes, after appearing to have subsided favorably, it relapses with suddenness and violence, or a metastasis to the meninges and substance of the brain takes place, which soon terminates in comatous or delirious death.

As to that variety which attacks infants a few days or weeks old, and is almost sure to prove fatal on account of its rapid spread from the point of its visible origin, over the whole body, the experienced Dr. Underwood is of opinion that its fatality is owing to particular circumstances, such as ill-ventilated chambers or crowded hospitals. I have, however met with several cases of infantile Erysipelas, where the circumstances mentioned did not in the slightest degree exist.

Dr. Good is of opinion that "what is usually called the infantile Erysipelas, is more commonly a variety of gangrenous erythema, produced in many instances by want of cleanliness, of pure air, and of nutritious food." However this may be, on a general scale, it has so happened that four cases which I have witnessed, were encountered in families who possessed and strictly observed all the conveniences and attentions desirable, in relation to food, ventilation, and cleanliness; so that unless these cases were accidental exceptions, the Doctor's opinion is gratuitous. From post-mortem examination of two infants who died of the disease, there seemed to have existed an affection closely resembling peritonitis, the whole surface of the abdominal parietes, and of the intestinal convolutions, having been covered with a serous fluid highly tinged with bile. If my recollection do not deceive me, my friend Dr. Eberle was present at one of the examinations, and gave his opinion that Erysipelas of infants a few days or weeks old is always sympathetic with intestinal disease. It seems probable, that in this variety the secretion of bile is naturally affected, since, in the cases submitted to my inspection, the specific eruption was of a much yellower tinge than in ordinary adult cases.

Having briefly stated symptoms, I pass on to the *prognosis* by which I mean a judgment of its *event*, for of its causes I have already spoken.

Erysipelatous inflammation, when superficial, and of moderate extent, unaccompanied by fever or much constitutional derangement, generally runs its course in from four to eight days, terminating favorably by desquamation. But when the disorder penetrates the skin and besets the cellular tissue, it is apt to become extensive and serious. On the extremities, more frequently than on the face or body, does this state of things occur, and the more serious and troublesome in proportion to its extent or spread, or as the rete mucosum is more or less affected.

When the face is attacked, the febrile symptoms which preceded the eruption seldom remit, but seem rather to increase with the increase of inflammation, and do not cease for a week or more, during which time the patient is in imminent danger from the violent delirium or coma so very prone to supervene, and plainly indicating the extension of diseased action to the surface or even parenchyma of the brain, while the skin continues very red and painful. If this state of things continue beyond the 8th day without terminating in resolution, we may expect suppuration to follow, which is known by a throbbing or pulsating sensation, superceding the burning pain now diminished. There is also at this time a diminution of the redness, as well as the chills; a suppuration, if it may be so called, takes place, and portions of cellular membrane are thrown off with the discharged pus. If, however the matter do not discharge in this way, as by ulceration, it is apt to remain a long time in the cavities, and to occasion great pain in the fasciæ, and even death from excessive irritation.

Œdematous swellings accompanying this form, enhance the danger of the patient. Acute diseases of the thorax or any of the abdominal viscera, very much dispose to a fatal termination. No external symptoms are more alarming than the degeneracy of the inflammation into gangrene, because this plainly indicates a prostration of the system, from which it can rarely be recovered. In general terms we may add, relative to prognosis, that if a cachectic patient be attacked, his chance of a favorable issue is but slender; if a part highly sensible be invaded, and if much fever, inflammation, or delirium attend, especially early in the disease, the patient is in great danger. A metastasis to the brain, lungs, abdominal viscera, or parietes, is very unfavorable. A translation of this kind I have no doubt of having arrested in myself; it was indicated by pain of the surface of the brain, and *incipient* delirium. In this condition I took at a dose two grains of opium, which in 30 minutes quieted the cerebral agitation, and induced a sound tranquil sleep, from which I awoke refreshed and convalescent.

There is sometimes great sensorial and nervous irritability, latent as it were, in erysipelatous disease of the face and head: of this a remarkable example presented in my own case. It was on the 7th day from the onset; I had retired to bed under the full persuasion that I was convalescing, and had enjoyed a quiet sleep of about three hours, when I was roused by a violent thunder, about 1 in the morning and after some time felt a chill, and irresistible stretching, followed soon after by violent arterial action, the pulse at about 130, the carotids beating with such force as apparently to cause my head to bounce from the pillow. An acute pain affected my left frontal sinus and temple, and diffused itself obscurely over the hemisphere of the same side. Four hours elapsed, when my pulse, reduced in frequency to about 90, was full and tense: my tongue thickly coated with a white fur; the pain of the left side of the head continued, the left eyeball was tender throughout its texture, and inclined to the copious emission of tears from the effort in writing a memorandum of my feelings. But ere the day closed, all disagreeable symptoms vanished in consequence of aperient medicines, and restoration to health rapidly followed.

I am aware that in reference to the Prognosis, I might be much more diffuse, but not in my opinion more edifying. It is not mine to display that happpy result of persevering study, and those wondrously retentive powers of memory, for which some of my brethren are so eminently conspicuous. To these, either from natural incapacity, or (what my self estimation would judge more probable,) from a concurrence of uncontrolable causes of an adverse nature, I am privileged to make very slender pretensions. My decided opinion, notwithstanding, is that Essays for discussion in a Medical Society, as well as observations made thereon, should be divested of prolixity, in order to leave room for discussion by the members generally, and consequently for a richer contribution of *experience* and *rational theory*, than can reasonably be expected, were the remarks of two or three individuals, exclusively, to occupy the time and attention.

It remains for me to treat of the *Methodus Medendi*, or Method of cure, which, in still more technical language, bears the title of *Therapeia.*

The general plan of cure is indisputably antiphlogistic, admitting of modification according to the type of the disease. — In plethoric or robust constitutions, when inflammatory symptoms are manifested in a high degree, when fever of the synochal character prevails; experience warrants general venesection, cathartics, principally saline, antimonial diaphoretics, &c. But when of mild character, or when the febrile condition is measurably overcome, local applications of sedative and discutient virtue should be recurred to and sedulously used. Of these, I have found a mixture of 3 parts of water of Acetate of Lead, one part of Camphorated Spirit, and half a part of Laudanum, to be most efficacious.

Should the inflammation resist this remedy after a fair trial, I apply an Epispastic over the whole affected part. This remedy, which, if I mistake not, was first suggested and used by our illustrious Physick, is one which when seasonably applied, seldom fails to arrest the progress of the disease, and to subdue its violence. A new action is thereby substituted, which commonly obliterates that of the disease, and the artificially vesicated part is then to be dressed either with Basilicon or Mercurial

Ointment. Even when the disease violently attacks the face, there need be no hesitation in applying the Cantharides Plaster, on the score of disfiguring the part, or causing unseemly scars. Thrice have I suffered with Erysipelas of the face and head—thrice smarted under vesicatories; and yet, as you perceive, Mr. Chairman, the delicate cuticle is restored in wonted smoothness, and my *beauty* continues unimpaired!

In some instances, where, before I saw the patients, Erysipelas had wandered round the body in a girdle of vesicles, I have followed it, *pari passu*, with the blistering plaster, and with the desired success. I rely therefore principally on this remedy, although prepared to admit the efficacy of Mercurial Ointment alone, in some cases, having used it over very extensive wanderings of the efflorescence, with most satisfactory result, and entertained no apprehensions about exciting ptyalism in my patients. Indeed, I have never known it to follow the extensive use of the blue ointment in the cure of Erysipelas.

From the experiments of Lawrence, Hutchinson, Dobson and others, the practice of incision and puncturing over the affected surface, claims respectful attention. It is new, but doubtless promises to become powerfully auxiliary in the treatment of this complaint, which is always troublesome, often treacherous, and sometimes suddenly fatal.

When the efflorescence appears in a mild form, unaccompanied by much fever, a cooling saline purge with a moderately antiphlogistic regimen, and the free use of the lotion above recommended, rarely fail, even without venesection or blistering, to overcome it. Much relief is also experienced by dressing the affected part with the farina of rye or buckwheat.

Of leeching, as a topical remedy, I can say little from experience, but since European practitioners of respectability have advantageously tested this remedy, there is reason to believe it safe and efficacious after general depletion.

When the muscular fasciæ have been penetrated by Erysipelatous inflammation, and suppuration has supervened, an outlet for the pus should be artificially made either by incision with the lancet, or by the introduction of a seton: Not only much suffering, but also destruction of parts, is obviated by this method. In one case under my care, I used the seton and bandage the former acting as a drain to the pus, while the latter approximated the parietes of the sinuous ulcers and promoted their adhesion.

If gangrene be the unhappy result of this inflammation, the fermenting poultice is probably the best application, and internally the bark, wine, and other stimulants are to be promptly administered.

Would it not be advisable, in cases where this troublesome and dangerous affection has made frequent and periodical inroads, to open an issue in some part of the body? It is presumable that this would operate as an efficient preventive of a return.

In conclusion it is proper to remark, that when Erysipelas attacks persons constitutionally debilitated, or if it be accompanied with typhoid symptoms; the tonic treatment is imperiously called for, aided by stimulants: of these, the bark in every form, and the volatile alkali, are to be preferred.

